# Prevalence of JC Polyomavirus in Patients with Neuroinvasive Disease of Unknown Etiology in Croatia

**DOI:** 10.3390/medicina60010069

**Published:** 2023-12-29

**Authors:** Tatjana Vilibic-Cavlek, Maja Bogdanic, Tajana Peric, Leona Radmanic, Ljiljana Antolasic, Ljiljana Milasincic, Snjezana Zidovec-Lepej

**Affiliations:** 1 Department of Virology, Croatian Institute of Public Health, 10000 Zagreb, Croatia; maja.bogdanic@hzjz.hr (M.B.); ljiljana.antolasic@hzjz.hr (L.A.); ljiljana.milasincic@hzjz.hr (L.M.); 2 School of Medicine, University of Zagreb, 10000 Zagreb, Croatia; 3 Department of Immunological and Molecular Diagnostics, University Hospital for Infectious Diseases “Dr. Fran Mihaljevic”, 10000 Zagreb, Croatia; tajanaperic.purs@gmail.com (T.P.); leona.radmanic@gmail.com (L.R.); szidovec@gmail.com (S.Z.-L.)

**Keywords:** JC polyomavirus, neuroinvasive disease, JCPyV DNA, JCPyV IgG antibodies, Croatia

## Abstract

*Background and Objectives*: John Cunningham polyomavirus (JCPyV) is a highly prevalent virus in the human population. The prevalence of JCPyV in patients with central nervous system disorders has not been examined extensively. The aim of this study was to analyze the prevalence of JCPyV DNA/antibodies in patients with neuroinvasive diseases (NID) of unknown etiology. *Materials and Methods*: The study included 132 patients with NID (febrile headache, meningitis, encephalitis) tested from January 2021 to December 2022. The control group consisted of 47 asymptomatic individuals. In patients with NID, serum and cerebrospinal fluid (CSF) samples were collected in the acute phase of the disease. CSF samples were tested for JCPyV DNA (PCR), while serum samples were tested for JCPyV IgG antibodies (ELISA). In controls, serum samples were tested for JCPyV IgG antibodies (ELISA). *Results*: JCPyV DNA was not detected in any of the CSF samples from patients with NID. JCPyV IgG antibodies were detected in 88.6% of patients and 74.5% of controls (*p* < 0.001). In the patients’ group, a significant difference in the IgG prevalence was observed between males (94.6%) and females (81.0%). In addition, significant differences in the seropositivity between age groups were found. The lowest seroprevalence (28.6%) was in patients less than 20 years, followed by a sharp increase in the 20–29-year group (69.2%), after which the seroprevalence remained stable (90.0–94.1%) in patients up to 69 years. All patients older than 70 years were JCPyV IgG-seropositive. No significant difference in the seroprevalence was found in patients presenting with febrile headache (81.6%), meningitis (93.3%), or meningoencephalitis (91.3%). No difference in the seropositivity between genders was found in controls. Although the seropositivity steadily increased in older participants, these differences were not significant. Analyzing the JCPyV antibody levels in patients with NID, the median antibody titers differed significantly between groups, ranging from 248 AU/mL (younger age groups) to 400 AU/mL (older age groups). *Conclusions*: Higher seroprevalence in the patients’ group highlights the need to further investigate the possible association of JCPyV and NID.

## 1. Introduction

John Cunningham or JC polyomavirus (JCPyV) was isolated in 1971 from the brain of a patient with Hodgkin’s disease [1]. JCPyV is a small, non-enveloped double-stranded DNA virus of the family *Polyomaviridae,* genus *Betapolyomavirus* [2]. The viral genome encodes regulatory and structural proteins including large T-antigen, small t-antigen, capsid proteins (VP1, VP2, and VP3), open reading frame (ORF1 and ORF2) [3,4], as well as an additional regulatory protein agnoprotein which is an important factor in viral DNA replication, transcriptional and post-transcriptional processes, and virus maturation [4]. The JCPyV genome contains the early and late transcriptional regions separated by a non-coding control region (NCCR) [2]. Based on the structure of NCCR, JCPyV has two distinct forms: the archetype and the neurotropic or prototypical form. The archetype has a highly conserved NCCR structure and is transmitted between individuals because it is the most common form in the environment. This form of the virus is not generally pathogenic. It is possible that neurotropic forms may develop in an individual through a process of neuroadaptation because their sequences are more variable. In the neurotropic form, extensive genomic rearrangements of the NCCR were found causing a high level of virus replication in the brain [5]. The prototype JCPyV exhibits strong tropism for glial cells, and replication of this strain in macroglial cells (mainly oligodendrocytes) causes white matter demyelination in the central nervous system (CNS) [6]. Neurotropic JCPyV can also infect astrocytes as well as cells of the choroid plexus and leptomeninges, which are important components of the blood–brain barrier, causing meningitis, meningoencephalitis, and meningeal syndrome. In addition, it is now known that JCPyV infects granule cells in the cerebellum, causing JCV granule cell neuronopathy, and cortical pyramidal neurons, causing JCV encephalopathy. The virus probably enters the site of initial intrathecal infection via hematogenous spread [7]. JCPyV has also been detected in various types of tumor cells of CNS origin, including primary CNS lymphoma [8]. Many studies have shown that JCPyV transformation of nonpermissive cells or cells that do not support JCPyV replication is a result of JCPyV-induced genomic instability. This instability may occur when JCPyV latently infected B lymphocytes leave reservoir sites such as the tonsils or kidneys and migrate through the bloodstream, where rearrangement of the NCCR occurs, or when infected cells enter germinal centers. JCPyV infection of glial cells is associated with DNA damage and chromosomal aberrations [9].

JCPyV is a highly prevalent virus in the world population. Seroprevalence studies indicate that seroconversion increases with age, and 40–80% of adult populations are seropositive in some regions [10,11,12]. JCPyV infection mainly occurs in childhood, likely via a fecal–oral route. Given the high prevalence of JCPyV in sewage and urine and the virus stability, it is possible that JCPyV spreads through polluted water, food, and fomites [13]. An aerogenic route of transmission is also possible, as JCPyV is commonly found in tonsil tissue and respiratory secretions [2]. While in some populations, most JCPyV infections are acquired in childhood, in others, JCPyV seroprevalence gradually increases, even in old age. These variations may indicate that JCPyV transmission depends more on cross-cultural differences or socioeconomic conditions. Anti-JCPyV antibody titers do not decrease with age, suggesting that JCPyV stimulation may continue throughout life, either through viral reactivation or reinfection. Reports of JCPyV DNA in peripheral blood leukocytes suggest the possibility of transmission by blood transfusion, and reports of the presence of JCPyV DNA in the seminal fluid also suggest the possibility of sexual transmission [14]. In addition, vertical transmission has been suggested, but there is no convincing evidence [2].

Primary infections are mainly asymptomatic, and the virus remains in an inactive latent state in the lymphoid tissue, bone marrow, kidneys, or brain for the remainder of the host’s natural life without causing disease [15]. Long-term immunosuppression induces the reactivation of JCPyV in the CNS, which can cause the potentially fatal syndrome known as progressive multifocal leukoencephalopathy (PML), a demyelinating disease of the CNS [7]. In addition, subclinical JCPyV reactivation occurs frequently in multiple sclerosis (MS) patients treated with natalizumab [16]. Moreover, rare cases of JCPyV-associated meningitis/encephalitis and nephropathy have been described [7,17,18]. JCPyV was the only pathogen found in the cerebrospinal fluid (CSF) of some patients with the characteristic meningeal signs and symptoms, according to several studies. However, it is unclear whether these cases result from primary JCPyV infection or reactivation [19]. In addition, it is not known how the immune system normally inhibits the replication and spread of JCPyV in the CNS and how compromised immunity may enable the spread of the virus through the CNS [20]. There is no evidence that the presence of JCPyV antibodies protects against future infection or viral reactivation [21].

The laboratory methods that are routinely used for the diagnosis of JCPyV include real-time polymerase chain reaction (PCR) and serology. The specificity of JCPyV PCR in the CSF is high (92–99%); however, the sensitivity varies (74–93%), therefore false negative results are possible [6,22]. Additionally, PCR only provides information about the presence of viral DNA and not about active viral replication. Although the virus can be isolated in primary cell cultures (embryonic cells) and peripheral blood mononuclear cells, routine cell culture for the diagnosis of JCPyV is not commonly performed in clinical laboratories [6]. The value of serology alone is limited because of the ubiquitous nature of the virus and a high proportion of IgG-seropositive individuals [9]. Neuropathological diagnosis of PML is often based on in situ hybridization (ISH) of JCPyV, which has proven to be more sensitive than immunohistochemistry (IHC) against JCPyV capsid proteins, but standard ISH methods typically only detect targets with moderate-to-high nucleic acid copy numbers [23].

Several (sero)prevalence studies on JCPyV were conducted in patients with PML [24] and MS [25,26,27]. However, the presence of JCPyV in patients with other diseases of the CNS has not been widely studied.

Because JCPyV PCR is not routinely performed in the CSF of patients with aseptic meningitis and unremarkable magnetic resonance imaging, the true prevalence of JCPyV meningitis is unknown [19]. In Croatia, data on the JCPyV are lacking. Only a few reports described PML in HIV-positive patients; however, data on the prevalence of this virus in other population groups are missing. This study aimed to analyze the JCPyV DNA and antibody prevalence in patients with neuroinvasive diseases of unknown etiology.

## 2. Materials and Methods

### 2.1. Patients and Controls

A total of 132 patients with neuroinvasive disease (febrile headache, meningitis, encephalitis) tested from January 2021 to December 2022 were included in the study. The viral etiology was suspected based on the CSF analysis (pleocytosis with mononuclear predominance, elevated protein level, and normal glucose level). None of the patients received medications that suppress the immune system such as immunosuppressive therapy and chemotherapy. CSF and serum samples were collected during the acute phase of the disease. CSF samples from all patients were initially tested for the most common neurotropic viral infections, including herpes simplex viruses (HSV-1/2), varicella-zoster virus, enteroviruses, neuroinvasive arboviruses: flaviviruses (tick-borne encephalitis, West Nile virus, Usutu virus), Toscana virus, Tahyna orthobunyavirus, and Bhanja bandavirus.

The control group included asymptomatic individuals who underwent a routine check-up (part of a physical examination, before orthopedic surgery, couples undergoing medically assisted reproduction). In controls (*n* = 47), serum samples were collected. 

In the patients’ group, there were 74 (56.1%) males and 58 (43.9%) females aged 9–93 years. In the control group, 23 (48.9%) participants were males and 24 (51.1%) were females aged 13–80 years. The median age (IQR) of patients and controls was 58 (IQR = 49–63) and 56 (IQR = 42–65), respectively (*p* = 0.142). Clinical presentations in patients with the neuroinvasive disease were febrile headache (49; 37.1%), meningitis (60; 45.5%), and meningoencephalitis (23; 17.4%).

### 2.2. JC Polyomavirus Antibody Detection

JCPyV IgG antibodies were detected using a commercial EIA (ELISA-VIDITEST anti-JCV IgG; Vidiac, Vestec, Czech Republic). In the solid-phase immunoassay used, the surface of the microplate wells is coated with a species-specific recombinant JCPyV antigen. Antibodies present in the test sample will bind to immobilized antigens. The bound antibodies then reacted in the next step with horseradish peroxidase-labeled anti-human IgG antibodies. The amount of bound-labeled antibodies was determined by a color enzyme reaction and read using a spectrophotometer at nm wavelength 450 nm and 620 nm reference filter within 10 min after stopping the reaction.

The antibody concentration was calculated using an e-calculator (www.vidia.cz, accessed on 23 September 2023) and expressed in arbitrary units (AU/mL). The results were interpreted as follows: AU < 28.0 negative; 28.0–35.0 equivocal; >35.0 positive. The manufacturer states a diagnostic sensitivity of 95% and specificity of 96%.

### 2.3. JC Polyomavirus DNA Detection

DNA extraction was performed by using the QIAAmp DNA Mini Kit (Qiagen, Hilden, Germany). Detection and quantification of JCV DNA was performed by using a standardized real-time PCR RealStar^®^ JCV PCR Kit 1.0 (Altona Diagnostics, Hamburg, Germany) on a Lightcycler 480 Instrument (Roche Diagnostics, Penzberg, Germany). The assay included a heterologous amplification system (Internal Control) to identify possible PCR inhibition and to confirm the integrity of the kit’s reagents. The probes specific for JCV DNA were labeled with the fluorophore FAM™ and the probe specific for the internal control with the fluorophore JOE™. Quantification standards in the assay (quantification range 10–10,000 JCPyV DNA IU/mL) were calibrated against the 1st World Health Organization International Standard for JCPyV for Nucleic Acid Amplification Techniques (NAT) (NIBSC code 14/114). 

### 2.4. Statistical Analysis

The differences in the seropositivity according to the patient’s demographic and clinical characteristics were compared using a Chi-square test. The differences in the antibody levels between sexes, age groups, and clinical diagnoses were assessed using a Kruskal–Wallis test. The strength of the association between dependent (JCPyV IgG seropositivity) and independent variables was assessed by logistic regression. Statistical analysis was performed using the Web Social Science Statistics program (https://www.socscistatistics.com/, accessed on 10 October 2023).

## 3. Results

The JCPyV DNA was not detected in any CSF samples. JCPyV IgG antibodies were detected in the serum samples in both tested groups. The difference in the overall JCPyV IgG prevalence among patients with neuroinvasive disease and controls was statistically significant (117/132, 88.6%, 95%CI = 81.1–92.9 vs. 35/47, 74.5%, 95%CI = 59.6–86.1, *p* = 0.019).

JCPyVV seroprevalence rates according to demographic and clinical characteristics of patients and controls are presented in Table 1.

In the patients’ group, a significant difference in the IgG prevalence was observed between males and females (94.6 vs. 81.0%, *p* = 0.014). In addition, there were significant differences in the seropositivity between age groups (*p* < 0.001). The lowest seropositivity was observed in patients less than 20 years old (28.6%), with a sharp increase in the 20–29-year age group (69.2%). A further progressive increase in the seropositivity was observed in the 30–39-year age group (90.0%), after which the seroprevalence remained stable at 90.9–94.1% in patients up to 69 years. All patients older than 70 years were IgG-seropositive to the JCPyV. Analyzing the seroprevalence according to clinical diagnosis, no significant difference in seropositivity was observed between patients with febrile headache (81.6%), meningitis (93.3%), or meningoencephalitis (91.3%).

In the control group, there was no significant difference in the IgG seropositivity among males and females (69.6 vs. 79.2%, *p* = 0.450). According to age, the seroprevalence varied from 62.5 to 100%. Although the progressive increase in seroprevalence was observed in older age groups up to 100% in patients older than 70 years, these differences were not significant (*p* = 0.157).

Analyzing the same patient and control age groups, a significant difference in the JCPyV seroprevalence was found only in the 40–49-year age group (94.1% in patients vs. 62.5% in controls, *p* = 0.044). The seropositivity in other age groups was also higher in the patients with neuroinvasive disease than in controls; however, these differences were not significant.

The results of the risk analysis showed that patients aged 20–29 years had almost eight times higher risk of being JCPyV-seropositive (POR = 7.87, 95%CI = 1.10–56.12; *p* = 0.039), while patients older than 30 years had PORs ranging from 31.50 to 201.00 compared to the youngest age group (≤19 years) (Table 2). Clinical presentation was not associated with a risk of being JCPyV-seropositive.

**Figure 1:**
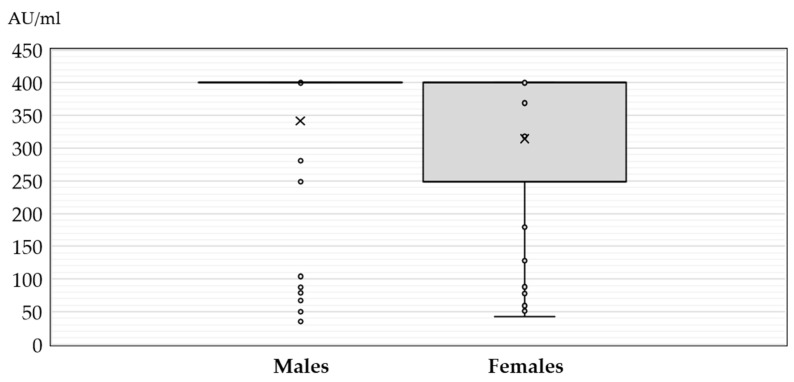
JC polyomavirus antibody titers (medians, interquartile ranges) in seropositive patients with neuroinvasive disease according to gender (× = mean values, o = inner and outlier points).

**Figure 2:**
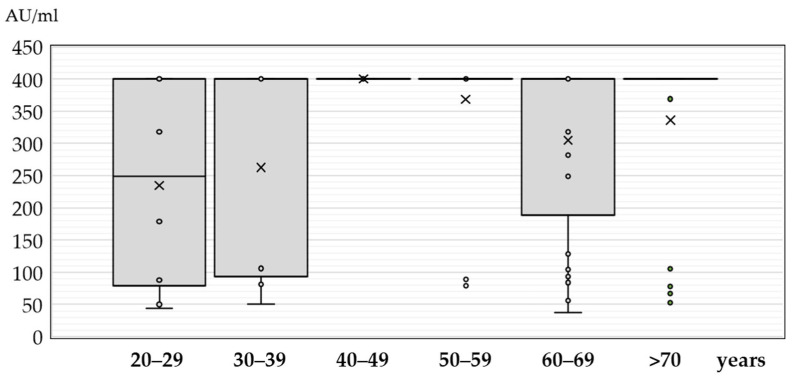
JC polyomavirus antibody titers (medians, interquartile ranges) in seropositive patients with neuroinvasive disease according to age (× = mean values, o = inner and outlier points).

**Figure 3:**
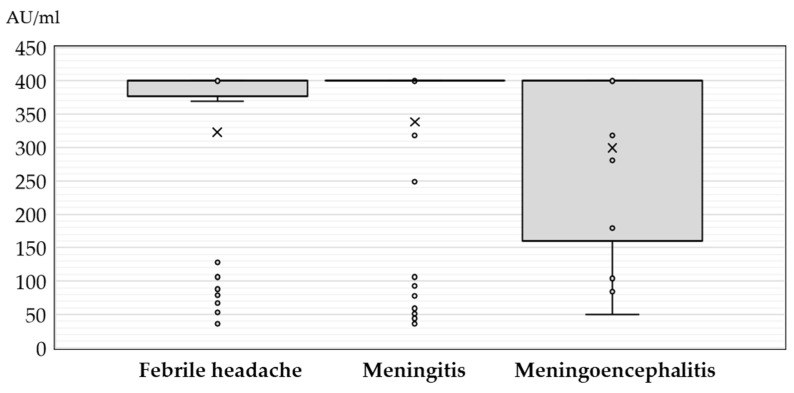
JC polyomavirus antibody titers (medians, interquartile ranges) in seropositive patients with neuroinvasive disease according to clinical diagnosis (× = mean values, o = inner and outlier points).

The JCPyV IgG antibody titers in patients with neuroinvasive disease according to sex, age, and clinical presentation are presented in Figure 1, Figure 2 and Figure 3. 

There was no significant difference in the antibody levels between seropositive males and females (median titer 400 AU/mL, IQR = 400–400 vs. 400 AU/mL, IQR = 248–400, *p* = 0.274) (Figure 1). 

The antibody titers differed significantly between age groups (*p* = 0.008). In the age group less than 20 years, only two seropositive samples showed an antibody titer of 179 AU/mL and 400 AU/mL. The median titers in patients older than 20 years ranged from 248 AU/mL (IQR = 69–400) to 400 AU/mL (IQR = 400–400) (Figure 2). 

No significant difference (*p* = 0.308) in JCPyV antibody titer was found in patients presenting with febrile headache (median 400 AU/mL, IQR = 369–400), meningitis (median 400 AU/mL, IQR = 400–400), and meningoencephalitis (median 400 AU/mL, IQR = 152–400) (Figure 3).

Analyzing the JCPyV antibody levels in the control group, no significant difference in antibody titers was found between males (median 400 AU/mL, IQR = 400–400) and females (median 268 AU/mL, IQR = 114–400; *p* = 0.308). In addition, no difference was observed between age groups (*p* = 0.467) with medians ranging from 168 AU/mL (IQR = 114–400) in younger individuals to 400 AU/mL (IQR = 400–400) in older individuals. 

Comparing the antibody titers in the same patient and control age groups, no significant difference in the median antibody levels was found between groups.

## 4. Discussion

The prevalence studies on JCPyV are mainly conducted among patients with PML and MS. However, the presence of JCPyV in patients with other pathological conditions of the CNS, including meningitis, has not been extensively studied.

JCPyV is not routinely tested in patients presented with meningitis; therefore, JCPyV-associated meningitis is rarely reported. In several published studies, JCPyV was detected in the CSF of both immunocompromised and immunocompetent patients with only meningeal symptoms [7]. In 1992, JCPyV infection associated with meningoencephalomyelitis in a non-immunosuppressed 13-year-old girl was described [28]. In 2005, a 38-year-old patient with long-standing systemic lupus erythematosus who presented with acute meningitis (fever, headache, and altered mental status) without encephalitis or PML was recorded [29]. JCPyV was the only pathogen found in CSF, suggesting a primary infection or symptomatic reactivation. The patient had no white matter lesions on magnetic resonance imaging, and her symptoms resolved spontaneously. Further, a fatal case of JCPyV aseptic meningitis in a 67-year-old HIV-seronegative patient was reported in 2014. The patient presented with the classic triad of cognitive impairment, gait disturbance, and urinary incontinence consistent with secondary normal pressure hydrocephalus [18].

A retrospective study from Finland (2012–2018) assessed the clinical indications and the impact of JCPyV DNA testing in clinical practice in a tertiary care center in Helsinki. Among patients with neurological symptoms suspected of PML, JCPyV DNA was detected in 4.9% of the CSF samples from patients with cognitive disorders, 5.1% with balance and gait problems, 19.0% with speech problems, and 5.3% with headaches [30].

Few studies have been conducted on the prevalence of JCPyV DNA in patients without neurological symptoms or in patients with neurological symptoms other than PML. One study conducted in 2003 analyzed the JCPyV DNA prevalence in CSF samples collected from South African (HIV-positive and -negative) and UK patients aged 16–68 years with suspected meningitis and encephalitis. While none of the UK patients tested positive, 2.8% of South African HIV-negative patients had detectable JCPyV DNA in the CSF samples [31]. In our study, none of the patients with neuroinvasive disease had detectable JCPyV DNA in the CSF samples.

It is unknown whether latent viruses such as JCPyV, which may be present in the brain, may be reactivated in the CNS during inflammatory processes caused by other viruses. A study from Sweden examined the CSF samples from patients with HSV-1 encephalitis, enteroviral meningitis, and meningitis of unknown etiology for the presence of JCPyV DNA, but no JCPyV DNA was detected in any sample, suggesting that other viral infections do not necessarily lead to JCPyV reactivation [32].

In the present study, in both the patient and control groups, high JCPyV seropositivity was observed. However, the prevalence of JCPyV IgG antibodies was significantly higher in patients with the neuroinvasive disease compared to asymptomatic individuals (control group; 88.6 vs. 74.5%). A lower, but not significant difference in seropositivity was observed in patients presenting with febrile headache (81.6%) than in patients with meningitis (93.3%) or meningoencephalitis (91.3%). Higher seropositivity in patients than in controls, especially in patients with meningitis/meningoencephalitis, suggests the possible role of JCPyV in the etiology of neuroinvasive infections.

In the published literature, the (sero)prevalence rates of JCPyV vary widely, depending on the region and studied group. A study conducted in Sweden analyzed the prevalence of JCPyV DNA in the CSF and blood of two cohorts of patients (patients with MS and other neurological disorders). Low JCPyV viral loads were detected in 0.5% of patients with multiple sclerosis, whereas JCPyV DNA was not detected in any CSF samples from patients with other neurological disorders. In addition, none of the patients in both groups had detectable JCPyV DNA in plasma samples. JCPyV antibodies were detected in 69% of plasma samples at moderate-to-high titers, with no difference between groups [25]. The other multicenter study conducted among MS patients in Canada, Europe, and Australia showed an overall JCPyV IgG seroprevalence of 57.1%, ranging from 48.8% to 69.5% [26]. In a Finnish study in the MS patient cohort, an overall JCPyV seroprevalence was 57.4%. The seropositivity was higher in men (67%) than in women (54%) and tended to increase with age, from 47% in patients less than 30 years to 79% in patients above 60 years [27]. In a multicenter Spanish study of MS patients, the JCPyV seropositivity was 55.3%, similar across regions, and increased significantly with age [33]. A higher seropositivity of 80% was found in Chinese MS patients [34].

Some other seroepidemiological studies conducted in the general population and blood donors showed an overall JCPyV IgG seropositivity of 57% in the Czech Republic [35], 58% in Switzerland [36], 62–69% in Queensland, Australia [37], and 91% in Portugal [38]. A Finnish study analyzed the JCPyV prevalence in pregnant women and their male spouses. The seroprevalence was 59.0% in women and 65.9% in men [39]. 

In a more recent study among Japanese patients with neuromyelitis optica spectrum disorders, the prevalence of JCPyV IgG antibodies was 80.8% [40].

In the present study, JCPyV IgG seroprevalence significantly increased with age. A sharp increase in seropositivity was observed from 28.6% in patients less than 20 years to 90.0% in patients in patients aged 30–39 years, with a slight continued rise throughout life up to 100% in patients older than 70 years. A steady increase in seropositivity with age was also observed in the control group, but these differences were not significant. Comparing the seroprevalence rates in the same age groups of patients and controls older than 30 years, seropositivity was higher in patients (90.0–100%) than in controls (62.5–100%), but significant differences were only in the 40–49-year age group (94.1% in patients vs. 62.5% in controls).

Similarly, in the abovementioned multicenter study, seroprevalence was significantly associated with age and gender. The anti-JCPyV antibodies prevalence increased with age, from 47.4% in patients aged 15–29 years to 64.1% in those aged 60 years and older, and was lower in women (55.3%) compared to men (61.6%) [26]. A progressive increase in the seroprevalence was also observed in Italy. In children less than 10 years, the seroprevalence was 9.5%, with the first rise in the 20–29-year age group (50.0%) and the second rise in the 40–49-year group (68.8%). Thereafter the seropositivity gradually increased up to 80.0% in individuals above 70 years [41]. In contrast, in an Australian study, age-specific JCPyV seroprevalence was up to 59.7% in the age of 50 years, 68.5% in people aged 50–70 years, and 63.6% in those aged over 70 years, but these differences were not statistically significant [37]. In a study from Cyprus, the seroprevalence curve was reverse U-shaped with higher seropositivity in patients aged 40–49 years (57.4%) than in patients younger than 40 years (35.6 and 41.9%, respectively) and patients of 50 years and older (47.4 and 36.4%, respectively) [42].

Similar to the high JCPyV IgG seropositivity detected in our study, a high seroprevalence was observed in Italian children aged up to three years. The overall seropositivity was 71.8%. The seroprevalence increased over time from 46.1% in one-month-old children to 80.7% in 12-month-old, 85.9% in 24-month-old, and 85.5% in 36-month-old children. One-month-old children were largely JCPyV IgM-negative (82.4%), and 58.8% of children developed IgM antibodies within the second and sixth months of life, suggesting that primary JCPyV infections likely occur in the first six months of age [43].

In our study, significant gender differences were found in patients with neuroinvasive disease with a higher seroprevalence in males than in females (94.6% vs. 81.0%). A similar gender association with JCPyV seropositivity was observed in Finland (men 67%, women 54%) [27]. In one Portuguese study, a slightly higher, but not significant difference in the JCPyV seropositivity was observed in females (93 vs. 88%) [38]. In the other study from Portugal, the JCPyV seroprevalence was slightly higher in males (65.3% vs. 58.7%); however, these differences were not significant [44]. No significant difference in the JCPyV seroprevalence between genders was also found in the Czech study (males 57.8%, females 55.4%) [35].

Analyzing the JCPyV IgG antibody levels of seropositive patients in our study, a significant difference in the antibody level was observed with higher levels in patients older than 40 years compared to the younger age groups. Higher antibody levels in older patients could be explained by more frequent JCPyV reactivations throughout life. 

In a Czech study, the levels of JCPyV antibodies in seropositive samples were stable among different age groups [35]. In a Polish study, the youngest MS patients (18–29 years old) had the lowest level of JCPyV antibodies, which increased over time up to 60 years old, although these differences were not significant [45]. 

In a study from Cyprus, there was no difference in the antibody levels between genders [42]. Similarly, our study found no significant difference in the antibody titers among males and females. 

The present study has some limitations that need to be addressed. The study included a small number of participants, and CSF samples were tested only for JCPyV DNA (not antibodies). These limitations should be mentioned when interpreting the results.

## 5. Conclusions

Detection of high seropositivity rates indicates that JCPyV is widely distributed in Croatia in both patients with neuroinvasive diseases and asymptomatic individuals. Although JCPyV DNA was not detected in any of the CSF samples, a significantly higher IgG seropositivity in both patients with meningitis (93.3%) and meningoencephalitis (91.3%) than in the asymptomatic individuals (74.5%) highlights the need for further investigation of a possible association between JCPyV and neuroinvasive diseases. Furthermore, it should be emphasized that the presence of JCPyV DNA or antibodies does not necessarily indicate active disease. Assessing the involvement of JCPyV in neuroinvasive disease requires a comprehensive clinical evaluation, including analysis of clinical data, symptoms, and specific laboratory results. Because JCPyV can access the brains of healthy people, the pathogenetic mechanism by which the virus reactivates in the CNS of immunocompromised individuals and becomes pathogenic remains to be further studied [46]. Healthcare professionals should be aware that JCPyV infection can cause clinically distinct entities and consider including this virus in the differential diagnosis of neuroinvasive disease, especially in immunocompromised patients.

## Figures and Tables

**Table 1:** Prevalence of JC polyomavirus IgG antibodies in patients with neuroinvasive disease and controls.

Characteristic	Patients				Controls			
	Tested *n* (%)	Positive *n* (%)	95%CI	*p*	Tested *n* (%)	Positive *n* (%)	95%CI	*p*
Sex				0.014				0.450
Male	74 (56.1)	70 (94.6)	86.7–98.5		23 (48.9)	16 (69.6)	47.1–86.8	
Female	58 (43.9)	47 (81.0)	68.6–90.1		24 (51.1)	19 (79.2)	57.8–92.9	
Age group				<0.001				0.157
≤19 years	7 (5.3)	2 (28.6)	3.7–70.9		1 (2.1)	0 (NA)		
20–29 years	13 (9.8)	9 (69.2)	38.6–90.9		2 (4.3)	1 (NA)		
30–39 years	10 (7.6)	9 (90.0)	55.5–99.7		8 (17.0)	5 (62.5)	24.5–91.5	
40–49 years	17 (12.9)	16 (94.1)	71.3–99.8		8 (17.0)	5 (62.5)	24.5–91.5	
50–59 years	22 (16.7)	20 (90.9)	70.8–98.9		9 (19.2)	6 (66.7)	29.9–92.5	
60–69 years	30 (22.7)	28 (93.3)	77.9–99.2		12 (25.5)	11 (91.7)	61.5–99.8	
70+ years	33 (25.0)	33 (100)	98.4–100 *		7 (14.9)	7 (100)	59.0–100*	
Clinical presentation				0.144				NA
Febrile headache	49 (37.1)	40 (81.6)	67.9–91.2		NA	NA	NA	
Meningitis	60 (45.5)	56 (93.3)	83.8–98.1		NA	NA	NA	
Meningoencephalitis	23 (17.4)	21 (91.3)	71.9–98.9		NA	NA	NA	

NA = not applicable; * One-sided 97.5% confidence interval; NA = Not applicable.

**Table 2:** Risk analysis for JC polyomavirus IgG seropositivity in patients with neuroinvasive disease and controls.

Characteristic	Patients			Controls		
	POR	95%CI	*p*	POR	95%CI	*p*
Male vs. female sex (ref.)	4.09	1.23–13.63	0.020	0.46	0.13–1.68	0.244
Age group						
≤19 years	Ref.					
20–29 years	7.87	1.10–56.12	0.039	NA	NA	NA
30–39 years	31.50	2.34–422.31	0.009	Ref.		
40–49 years	56.00	4.33–724.08	0.002	0.66	0.07–5.87	0.715
50–59 years	35.00	4.11–297.68	0.001	0.80	0.09–6.84	0.838
60–69 years	49.00	5.83–411.42	<0.001	4.40	0.31–60.61	0.268
70+ years	201.00	8.71–4634.36	<0.001	6.81	0.26–172.29	0.244
Clinical presentation						
Febrile headache	Ref.			NA	NA	NA
Meningitis	3.15	0.90–10.94	0.070	NA	NA	NA
Meningoencephalitis	2.36	0.46–11.94	0.298	NA	NA	NA

POR = prevalence odds ratio; CI = confidence interval; NA = Not applicable.

## Data Availability

Data are contained within the article.
